# Scientific Items

**Published:** 1888-03

**Authors:** 


### SCIENTIFIC ITEMS.

**Great War Ships and Forts.**—Are armored ships and big guns and forts necessary to an effective defense? The Senate, in favoring a preliminary appropriation of $21,000,000, has virtually said yes. It remains for the House to put in its measure. Outside of Congress, there is quite as distinct difference of opinion in regard to the general proposition among the well informed. Those who do not favor the building of a great armored fleet and costly ship works have recently been joined by Captain John Ericsson, the designer of the famous Monitor.

He makes the point that a port like New York can be successfully defended without them and following this argument to its conclusion, those opposed to great outlays for ships and forts might logically insist that by Ericsson's admission they were unnecessary.

The problem before us is how to beat off a fleet of modern war ships, whose tactics during bombardment would be that of retreating to the open sea before night.

"I have for a series of years studied, under special advantages, the problem of defending the harbor of New York against first-class ironclad ships. I have positive grounds for recommending the adoption of the submarine gun of 16 in. caliber, as applied in the Destroyer. This gun possesses power and capacity to expel projectiles carrying explosive charges weighing 300 pounds, hence capable of scattering the hull of a Lepanto or an Inflexible. The vessel carrying the submarine gun, being protected by an impregnable breast-work of inclined solid armor plates two feet thick, backed by six feet of timber, is capable of resisting any ordnance whatever during attack bows on. I deem it important to observe that, like the Destroyer, all vessels carrying the submarine gun, whatever be their size, must be provided with steam turning gear, by means of which they can be directed to any point of the compass without backing or going ahead.—*Scientific American.*

**The Nose the Source of all our Woes.**—At the last congress of German naturalists and physicians, held in Wisebaden, Dr. Gracy reported several cases of mental disturbance characterized by an impossibility of fixing the attention on any subject, except for a very brief period, or of prolonged mental effort of any kind whatever. This condition, to which the author gave the name of aproxia, was always associated with certain lesions of the nasal mucous membrane and obstruction to the passage of air through the nasal fossæ.

This is, we believe, the latest accusation which has been brought against the sinful nose. Headache, cough, dyspnœa, earache, neuralgia, hay fever, acne, convulsions, and syncope are only a few of the many evils which this troublesome organ is accused of having inflicted upon long suffering man, and it bids fair to outstrip even the ovaries as a center for morbid reflexes. As regards aproxia, it is said not to be a reflex. and the mechanism of its production is assumed to be a purely physical one. The lymphatic spaces beneath the dura mater have been found to be in direct communication with the mucous membrane of nasal fossæ, and inflammation of the latter is supposed to interfere with the elimination of the waste products resulting from cerebral activity, thus leading to mental sluggishness. But whatever may be its methods, the nasal organ is evidently responsible for many, if not most, of our ills. Clearly, the nose must go.—*N. Y. Medical Record.*

**Solidification of Liquids by Pressure.**—Theoretically, the formula given by J. Thomson implies that solidification becomes possible at a given temperature and under sufficient pressure, provided the density be greater in the solid than in the fluid state. Practically, no instance is known of any liquid—properly so-called—being capable of solidification by pressure alone. At a recent meeting of the Paris Academy of Sciences, however, Mr. E. H. Amagat stated, that after a number of experiments, he has succeeded in solidifying the bichloride of carbon ($C_2 Cl_4$), obtaining some crystals which appear evidently to belong to the cubic system. This substance is solidified at the temperatures of—19.5°, 0°, 10°, and 19.5° C., under the respective pressures of 210, 620, 900, and 1,160 atmospheres. This and other results would seem to imply that every fluid has a critical point of solidification; that is, a temperature above which solidification will take place under no pressure: just as there appears to be a temperature below which the body remains solid under the slightest pressures.—*Popular Science News.*

**The** experiments of Nahrwold and other electricians seem to demonstrate that the atmosphere, when free from foreign matter, cannot hold electricity nor serve as a conductor. The floating particles of dust are, it would appear, an important element in the phenomena of a thunder-storm, the passage of which always leaves the air so clear and clean. Audries's theory of the peculiar danger from lightning in a manufacturing town, with its smoke-laden atmosphere, would thus be scientifically verified.—*Ib.*

**What** is the difference between a tornado and a cyclone, and from what authorities? The word tornado is used to indicate any wind of extreme violence, from 90 to 120 miles an hour. The word cyclone is properly used to denote whirlwinds, which in the northern hemispheres rotate in directions opposed to that of the hands of a watch. The Cyclopedias, Haswell, and Ganot all speak of the subject.—*Scientific American.*

**The Chrotograph** is a pencil manufactured in Germany for writing on the skin. It is made in various colors, and affords legible writing, which can be easily removed without the use of water. It is designed for the use of physicians, to make memoranda upon their patients.—*Ib.*

